# Age-stratified assessment of orthodontic tooth movement outcomes with clear aligners

**DOI:** 10.1186/s40510-024-00542-2

**Published:** 2024-11-11

**Authors:** Mariamma Chaluparambil, Sarah Abu Arqub, Chia-Ling Kuo, Lucas Da Cunha Godoy, Madhur Upadhyay, Sumit Yadav

**Affiliations:** 1 University of Florida, Gainesville, USA; 2 University of Jordan, Amman, Jordan; 3 University of Connecticut Health Center, Farmington, USA; 4 University of Nebraska Medical Center, Omaha, USA

**Keywords:** Clear aligners, Adults, Teenagers, Accuracy

## Abstract

**Objective:**

This study compared the achieved tooth movement to that predicted in the preliminary simulated digital treatment plan between adults and teenagers.

**Materials and methods:**

Records of 60 patients (30 adults; mean age: 36.6 ± 11.36 years, and 30 teenagers; mean age: 16.23 ± 2.25 years) were randomly selected. Initial and predicted models were obtained from the initial simulated treatment plan. The first model of the refinement scan was labeled as achieved. SlicerCMF software (version 3.1; https://www.slicer.org) was used to superimpose the achieved and predicted digital models over the initial ones with regional superimposition on relatively stable first molars. 600 teeth were measured in each group for horizontal, vertical, angular movements, and transverse dimensions. Accuracy was defined as the achieved amount of movement minus predicted and was calculated for each individual and compared between teens and adults.

**Results:**

The mandibular inter-canine width accuracy was statistically significant between groups (*p* = 0.050). Significant under-correction in horizontal movements was noted for mandibular first premolars in teenagers compared to adults (*p* = 0.037). There was considerable over-correction in horizontal movements for mandibular central and lateral incisors between groups (*p* < 0.05). No significant difference was observed between groups in the vertical plane. Rotations were underachieved for maxillary first premolars, more in adults than teenagers (*p* = 0.017).

**Conclusion:**

The accuracy of achieved versus predicted tooth movement between adults and teenagers was significant for the inter-canine width. Mandibular central and lateral incisors showed significantly greater over-correction in adults in the horizontal plane. The accuracy of rotations and vertical movements was comparable.

**Supplementary Information:**

The online version contains supplementary material available at 10.1186/s40510-024-00542-2.

## Background

Clear aligners evolved from Kesling’s 1940 positioner appliance for refining teeth [1]. With advancements in CAD/CAM technology, Align Technology^®^ introduced Invisalign^®^ in the late 1990s as the first esthetic alternative to fixed orthodontics [2]. Initially featuring “divots” for tooth pushing and “windows” for positioning, Invisalign^®^ later integrated attachments, buttons, and elastics to enhance efficacy [1, 3, 4]. Recent enhancements include optimized attachments for better control of tooth movements across all planes, placed automatically by the software [5].

Initially gained popularity among adult patients [6]. In recent years, due to marketing strategies, there has been an increased number of teenagers demanding clear aligner treatment for esthetics. Several studies have assessed orthodontic outcomes with clear aligners in adult populations with different malocclusions [7–9]. These studies have primarily indicated that the major concern with clear aligner’s treatment is the level of accuracy between the predicted and achieved clinical outcomes [4, 10–12]. Kravitz et al. (2009) reported a mean accuracy of 41% for the predicted tooth movement with Invisalign [12]. Additionally, a recent study reported an accuracy of 50%, with buccolingual movement being the most accurate (56%), while maxillary canine rotation (37%), mandibular first molar rotation (28%), and mandibular incisor intrusion (35%) were the least accurate [10]. Complex movements such as extrusion, torque, and rotations are challenging for clear aligner mechanotherapy [4]. Not surprisingly, tipping remains the most predictable tooth movement achieved with clear aligners [9]. Therefore, compared to fixed appliance treatment, some studies reported inefficiency in achieving similar treatment outcomes between clear aligners and fixed appliances [13, 14], while others showed comparable treatment outcomes between the two systems [15].

With Invisalign^®^ Teen gaining popularity, Borda et al. were the first to compare treatment outcomes between clear aligners and fixed appliances in teenagers with mild malocclusion using standardized indices [16]. Their results indicated comparable outcomes between the two appliances for marginal ridge positioning, buccolingual inclination, interproximal contacts, and occlusal contacts [16]. However, aligners achieved better results in alignment, overjet, and occlusal relationships, as well as more favorable outcomes regarding treatment duration, number of emergency visits, and overall appointments [16]. While their study focused on treatment outcomes and efficiency in mild malocclusions in teenagers, there is a lack of evidence regarding the predictability of achieving planned tooth movements in teens with clear aligners for various malocclusions. This is especially concerning given the increased marketing of clear aligners to younger age groups and the rising demand among teenagers. The bone architecture differs between adults and teenagers, potentially influencing bone modeling and remodeling in response to applied forces. This might affect treatment duration and clinical outcomes. However, no studies have compared the treatment outcomes and duration associated with clear aligners between adults and teenagers. Therefore, this pilot study employed up-to-date 3D model superimposition to compare achieved tooth movements with predictions from the initial simulated digital treatment plan between adults and teenagers. Our null hypothesis posited that the predicted tooth movement outcomes in teenagers are comparable to those in adults.

## Materials and methods

### Sample collection

The research protocol for this retrospective comparative study was reviewed and approved by the Institutional Review Board at the University of Connecticut Health (IRB#22X-197-2). Records of 60 patients (30 adults; average age: 36.6 ± 11.36 years, and 30 teenagers; average age: 16.23 ± 2.25 years) were selected for the study. Selection of the records comprised random screening of the successively completed clear aligners (Invisalign^®^) cases backward in time until 30 cases were selected for inclusion in each group.

The simulated treatment plan was developed by the same clinician, an expert in clear aligner therapy with over 20 years of experience. The clinician followed a standardized algorithm with no restrictions on interproximal reduction (IPR), which was performed as necessary, or on the placement of attachments. Aligners were changed weekly, and the average treatment time was (36.6 ± 11.36 months in adults and 16.23 ± 2.25 months in teenagers). Patients included in the study started treatment in 2021 and later, after the introduction of SmartTrack Invisalign^®^ material. The included subjects met the following inclusion criteria: (1) Subjects between 13 and 19 years old for the teens group, and above 20 years old for the adult group with a full permanent dentition, (2) Angle Class I, mild Class II, and Class III cases with a non-extraction treatment plan, featuring mild to moderate crowding (2–6 mm) or spacing (1–6 mm), (3) No skeletal transverse constriction, (4) Both arches were included in the treatment, (5) Patients who completed the first stage of their Invisalign^®^ treatment and had a refinement scan with an acceptable field of view for all teeth, (6) Charts indicated consistent compliance with appointments and aligner’s wear, (7) Clincheck^®^ simulated treatment plan had no movement of molars in any of the three planes of space. Patients were excluded if: (1) Charts indicated non-compliance and inconsistency of treatment, (2) Patients had posterior scissor bite or crossbite malocclusion, (3) Missing teeth, (4) If movement was planned for molars according to the Clincheck^®^ software simulated plan, (5) Cleft lip and palate and other Craniofacial malformations, (6) Poor-quality stereolithography files (STL) files with inadequate field of view.

**Fig. 1:**
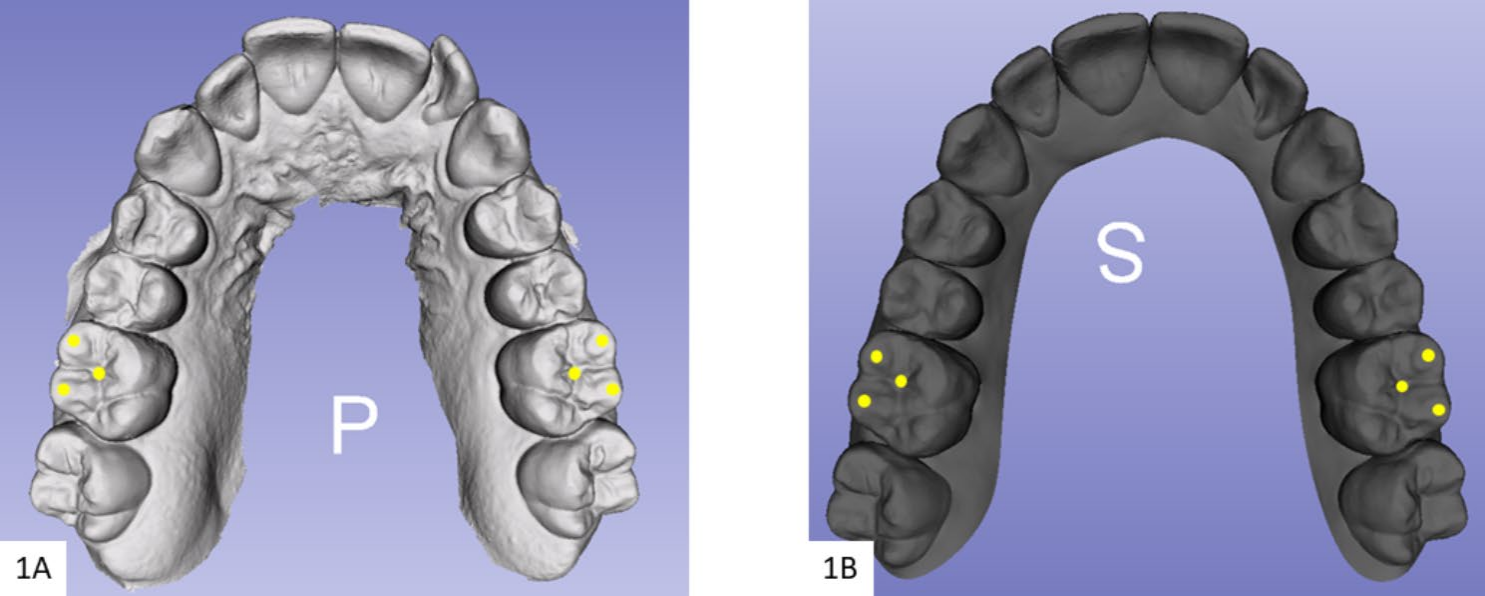
Superimposition using the buccal cusp tips and the center of the central fossa of the first molar. **1-A** initial digital 3D model. **1-B** predicted digital 3D model

**Fig. 2:**
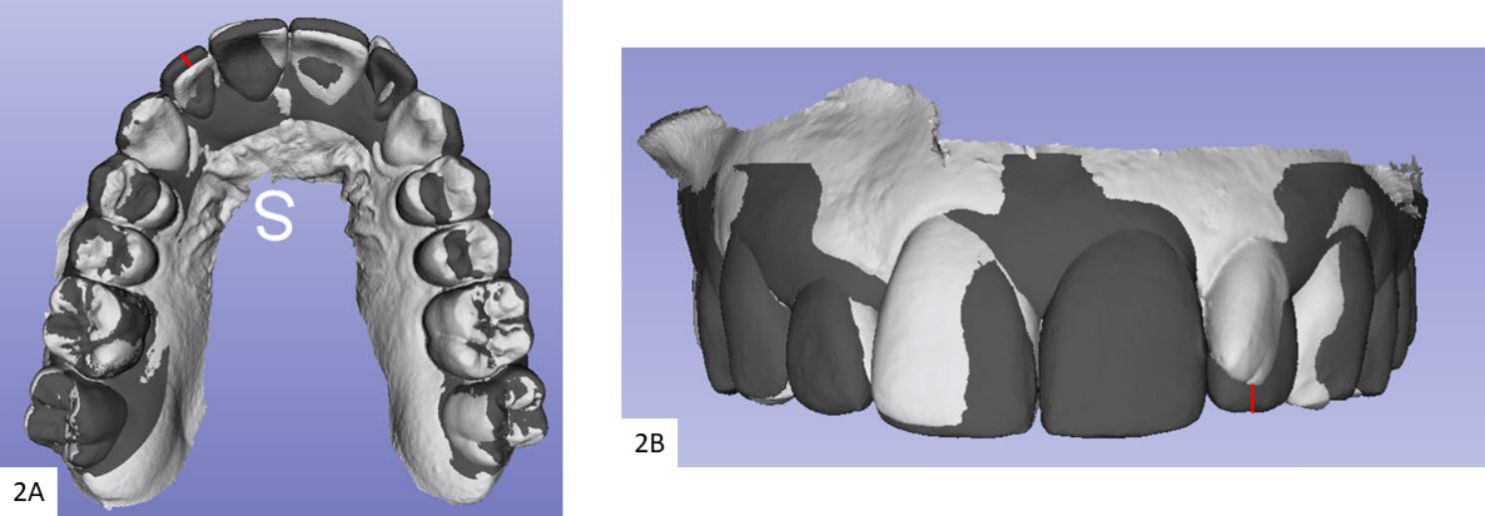
**2-A**: Horizontal movements measurement technique,**2-B**: Vertical movements measurement technique

**Fig. 3:**
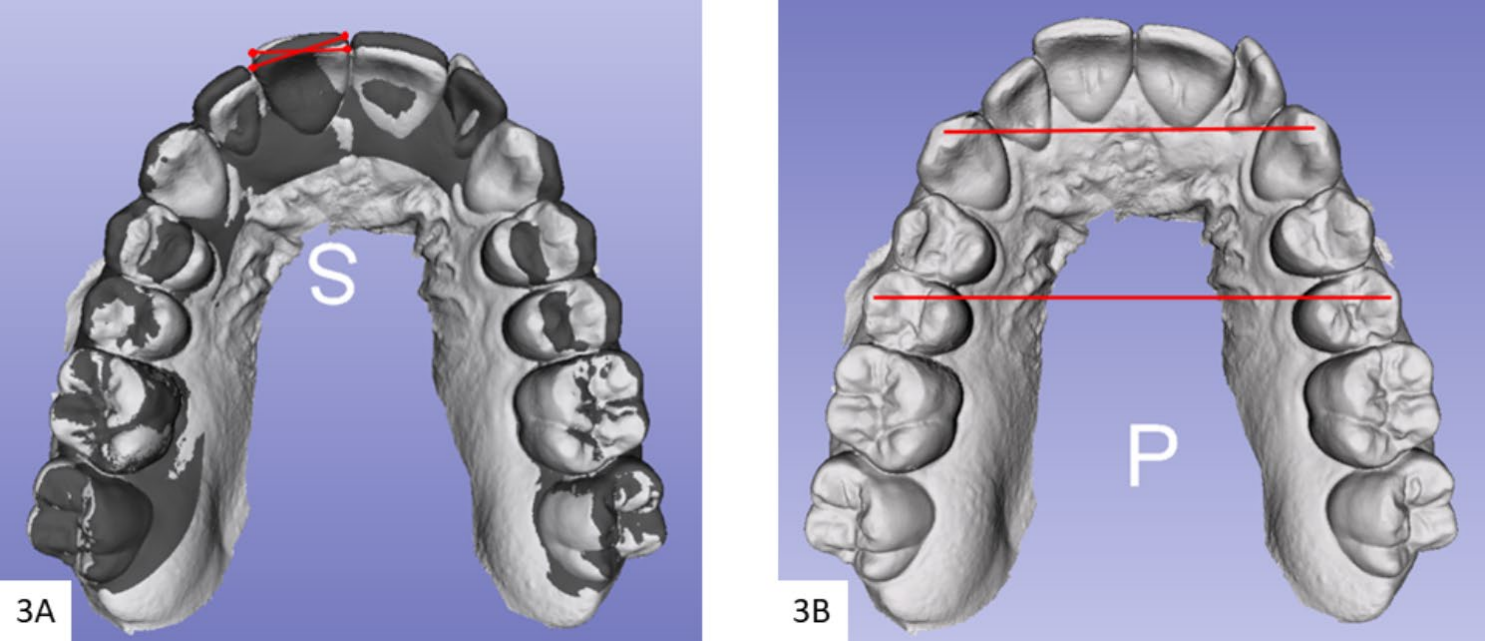
**3-A**: Mesiodistal rotations measurement technique, **3-B**: Inter-canine and inter-premolar widths measurement techniques

Three digital models for each included subject were exported as STL files from the Invisalign^®^ doctor’s account. The initial and final models from the first stage of treatment (the first simulated Clincheck^®^ treatment plan), were marked as initial and predicted accordingly. Further, the initial model for the refinement simulated treatment plan was downloaded from the Clincheck^®^ software and marked as achieved designating the definite results after wearing the first set of Invisalign clear aligners [8]. A calibrated examiner used SlicerCMF (open-source, version 3.1; https://www.slicer.org) software for the comparison between the models. Consequently, the predicted and achieved 3D dental models in both groups were superimposed over the initial, using the regional superimposition technique on the molar teeth that seemed proportionally stable [8, 17]. The central fossa and buccal cusp tips of maxillary first molars were selected as points for the superimposition (Fig. 1). The calculated amount of predicted and achieved movements in each group was assessed on the superimposed initial over predicted, and initial over-achieved models in succession, and eventually compared. No superimposition was done for the achieved model over the predicted. The overall number of teeth included in the measurements was (1200) for both adults (600) and teens (600). Teeth included in the measurements were: maxillary and mandibular central incisors and lateral incisors, canines, first premolars, and second premolars. This methodology has been extensively validated by us and other authors [8, 18]. The extent of achieved movement was calculated by pointing out the change in the position of teeth from the initial to the achieved virtual dental model (AVDM). And that predicted was calculated by pointing out the change in the position of the teeth from the initial to the predicted virtual dental model (PVDM). To assess the horizontal movements, the models were viewed from the occlusal plane, and the horizontal variance between the models was calculated from the center of the buccal cusp tips or the incisal edges with the 3D Slicer software ruler (Fig. 2-A). Similarly, the same anatomical points were used to assess the vertical discrepancies but from the labial or buccal views (Fig. 2-B). Mesiodistal rotations were assessed in the horizontal plane by angles formed between the lines on each set of superimposed models, of which each line connected 2 points on the labial and lingual cusp tips of premolars, mesial and distal points on the incisal edges of the incisors and the occlusal surface of the canines (Fig. 3-A). Finally, the distance between the cusp tips of the canines and premolars was used to tabulate the inter-canine and inter-premolar widths respectively (Fig. 3-B). Additionally, data with regard to treatment duration and number of refinements in each group were also collected from the charts.

Accuracy was defined as the achieved amount of movement minus the predicted:

### Accuracy = achieved – predicted

The accuracy was calculated for each individual and compared between teens and adults. To assess intra-examiner reliability, remeasurement for randomly selected 10 subjects (5, adults and 5 teens) was done one month after the initial measurements, by the same examiner.

### Statistical analysis

Demographic variables, predicted, and achieved movements were summarized descriptively for adults and teens separately. Left and right movements were averaged before analysis. Accuracy was assessed against perfect accuracy (accuracy = 0) using a one-sample t-test within groups and compared between teens and adults using a two-sample independent t-test and a linear regression model adjusting for predicted movement. These analyses were conducted by arch, tooth, and plane. All statistical tests were two-sided, with significance set at *p* < 0.05. Statistical analyses were performed using R version 4.1.2.

### Posthoc power analysis

Based on a posthoc power analysis performed in G*Power 3.1.9.7, assuming normal distribution and a common variance of accuracy between adults and teenagers, a sample size of 30 adults and 30 teenagers would provide 81% power to detect a mean difference of 0.75 standard deviations in accuracy between the two groups, at a 5% significance level, using a two-sided independent t-test.

## Results

### Demographics and clinical characteristics

As indicated in Table 1, both groups were comparable in terms of gender, treatment duration, and number of refinements needed to complete treatment. The mean age of the adult group was 36.6 ± 11.36 years, while that for the teenagers was 16.23 ± 2.25 years.

**Table 1:** Clinical characteristics of included subjects

	All (*n* = 60)	Adults (*n* = 30)	Teenagers (*n* = 30)	*P*-Value
Sex				0.604
Female	27 (45%)	15 (50%)	12 (40%)	
Male	33 (55%)	15 (50%)	18 (60%)	
Refinements				0.847
1	20 (33.33%)	11 (36.67%)	9 (30%)	
2	20 (33.33%)	9 (30%)	11 (36.67%)	
3	9 (15%)	4 (13.33%)	5 (16.67%)	
4	7 (11.67%)	3 (10%)	4 (13.33%)	
5 or more	4 (6.67%)	3 (10%)	1 (3.33%)	
Age (years)	26.42 ± 13.09	36.6 ± 11.36	16.23 ± 2.25	< 0.001*
Treatment Duration (months)	23.93 ± 14.04; 21 (5, 69)	22.27 ± 16.09; 18 (5, 69)	25.6 ± 11.68; 22.5 (5, 63)	0.363

* <0.05

### Transverse plane

In within-group comparison, the deviation of achieved inter-canine or inter-premolar width from that of predicted was not statistically significant in each of the assessed groups, except for the mandibular inter-canine width that showed a greater average of achieved width (26.73 ± 2.1) compared to the predicted (26.38 ± 2.06) in the adult group (*p* = 0.039) (Table 2**).** When both groups were compared, the accuracy for the mandibular inter-canine width showed a statistically significant difference between groups (overcorrected in the adult group vs. under-corrected in the teenagers’ group) after adjusting for predicted movement (*p* = 0.050). Table 3.

**Table 2:** Descriptive statistics of transverse measurements’ changes in adults and teenagers

Arch	Width change	Predicted (*P*) mean ± standard deviation; median (minimum, maximum)	Achieved (A) mean ± standard deviation; median (minimum, maximum)	Accuracy (A-*P*) mean ± standard deviation; median (minimum, maximum)	*P*-Value
Adults					
Mandibular	Inter-premolar	40.93 ± 2.48; 40.73 (35.25, 46.05)	40.99 ± 2.76; 40.81 (36.09, 47.72)	0.06 ± 0.87; 0 (-1.97, 1.9)	0.707
	Inter-canine	26.38 ± 2.06; 26.12 (23.37, 30.53)	26.73 ± 2.1; 26.5 (23.55, 31.3)	0.35 ± 0.9; 0.28 (-2.45, 2.5)	0.039*
Maxillary	Inter-premolar	48.57 ± 2.54; 48.53 (42.93, 54.58)	48.05 ± 3.12; 48.29 (38.94, 55.07)	-0.52 ± 1.52; -0.42 (-6.92, 1.69)	0.072
	Inter-canine	35.67 ± 2.38; 35.52 (31.38, 40.52)	35.93 ± 3.31; 35.16 (30.94, 46)	0.26 ± 2.14; -0.23 (-2.12, 8.3)	0.506
Teenagers					
Mandibular	Inter-premolar	41.28 ± 2.59; 41.11 (35.88, 48.53)	40.87 ± 2.46; 40.55 (36.46, 48.35)	-0.41 ± 1.49; -0.17 (-3.92, 1.46)	0.144
	Inter-canine	27.2 ± 2.18; 27.02 (23.08, 32.29)	26.95 ± 2.15; 26.92 (23.22, 32.24)	-0.25 ± 1.08; -0.12 (-3.21, 1.3)	0.220
Maxillary	Inter-premolar	49.07 ± 2.91; 49.3 (41.73, 56.52)	48.77 ± 2.87; 48.93 (43.19, 56.48)	-0.3 ± 1.83; -0.18 (-4.74, 6.17)	0.372
	Inter-canine	35.44 ± 2.29; 35.45 (30.96, 40.96)	35.28 ± 2.27; 35.19 (30.89, 41)	-0.15 ± 0.66; -0.08 (-1.53, 1.45)	0.211

* <0.05

**Table 3:** Comparison between adults and teenagers in the accuracy of transverse measurements

					Adjusted mean accuracy difference comparing teenagers to adults			
Arch	Width change	Accuracy in adults	Accuracy in teenagers	*P*-value between groups	Estimate (beta)	95% CI lower bound	95% CI upper bound	*P*-value
Mandibular	Inter-premolar	0.06 ± 0.87; 0 (-1.97, 1.9)	-0.41 ± 1.49; -0.17 (-3.92, 1.46)	0.143	-0.44	-1.05	0.17	0.166
	Inter-canine	0.35 ± 0.9; 0.28 (-2.45, 2.5)	-0.25 ± 1.08; -0.12 (-3.21, 1.3)	0.023	-0.51	-1.02	-0.01	0.050*
Maxillary	Inter-premolar	-0.52 ± 1.52; -0.42 (-6.92, 1.69)	-0.3 ± 1.83; -0.18 (-4.74, 6.17)	0.623	0.26	-0.59	1.11	0.554
	Inter-canine	0.26 ± 2.14; -0.23 (-2.12, 8.3)	-0.15 ± 0.66; -0.08 (-1.53, 1.45)	0.315	-0.42	-1.23	0.39	0.319

*<0.05

### Horizontal plane

**Fig. 4:**
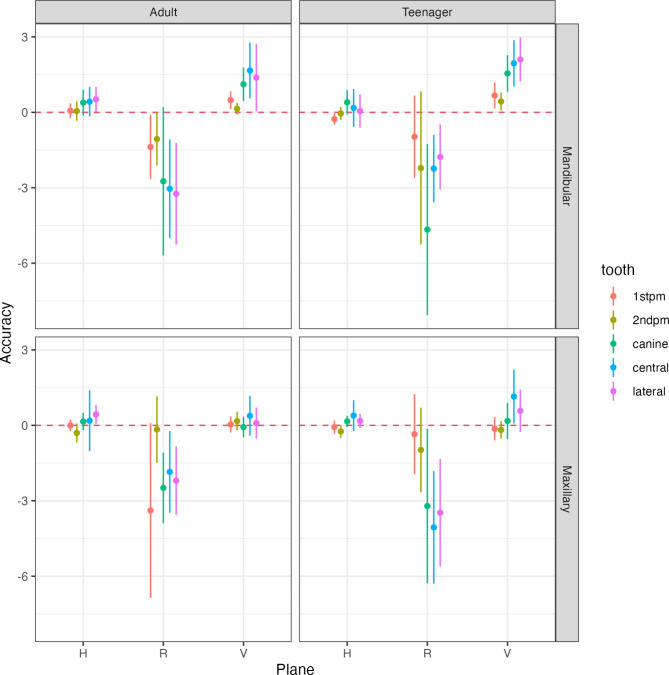
Descriptive statistics for the various predicted and achieved movements for adults and teenagers

The within-group comparison showed no significant difference between the achieved and predicted movements in the horizontal plane for the majority of the assessed teeth. However, the mandibular lateral incisors in the adult group showed significantly more (*p* = 0.042) achieved movement than predicted, whereas mandibular first premolars in the teenage group showed significantly less (*p* = 0.022) achieved movement than predicted. Figure 4 and Supplementary Table 1.

When both groups were compared, a significant under-correction (*p* = 0.037) in horizontal movements was noticed for mandibular first premolars in teenagers than in adults after adjusting for predicted movement. There was a significant over-correction (*p* < 0.05) in horizontal movements for mandibular central and lateral incisors in adults when compared to teenagers after adjusting for predicted movement. Table 4.

**Table 4:** Comparison between adults and teenagers in the deviation of the achieved tooth measurements from the predicted

						Adjusted mean accuracy difference comparing teenagers to adults				
Arch	Tooth	Type of movement	Accuracy in adults	Accuracy in teenagers	*P*-value between groups	Estimate (beta)	95% CI lower bound	95% CI upper bound	*P*-Value	
Mandibular	1stpm	H	0.06 ± 0.8; 0.03 (-1.25, 2.35)	-0.27 ± 0.6; -0.06 (-1.71, 0.52)	0.077	-0.34	-0.66	-0.03	0.037	
		R	-1.38 ± 3.45; 0 (-11.8, 3.65)	-0.97 ± 4.4; 0 (-14.1, 12.7)	0.692	1.21	-0.57	2.99	0.187	
		V	0.49 ± 0.95; 0.61 (-1.77, 2.16)	0.67 ± 1.4; 0.41 (-2.28, 4.04)	0.554	0.00	-0.43	0.44	0.986	
	2ndpm	H	0.05 ± 1.04; -0.01 (-3.71, 2.6)	-0.04 ± 0.68; 0 (-1.44, 1.72)	0.676	-0.28	-0.57	0.02	0.076	
		R	-1.06 ± 2.83; 0 (-7.5, 7.25)	-2.21 ± 8.15; 0 (-31.35, 9.75)	0.468	2.19	-0.10	4.49	0.066	
		V	0.14 ± 0.63; 0.05 (-1.02, 1.75)	0.43 ± 0.96; 0.36 (-1.42, 2.64)	0.174	0.13	-0.16	0.42	0.383	
	Canine	H	0.39 ± 1.37; 0 (-1.83, 5.28)	0.4 ± 1.3; 0 (-2, 4.06)	0.977	-0.17	-0.76	0.41	0.563	
		R	-2.74 ± 7.9; 0 (-33, 9.5)	-4.66 ± 9.12; -2.45 (-34.5, 6.1)	0.387	1.15	-1.55	3.86	0.407	
		V	1.12 ± 1.77; 0.95 (-2.42, 7.44)	1.55 ± 1.95; 1.13 (-0.92, 9.14)	0.373	-0.28	-1.05	0.48	0.472	
	Central Incisor	H	0.43 ± 1.57; 0 (-3.2, 4.57)	0.18 ± 2.02; 0 (-6.5, 4.31)	0.598	-0.74	-1.43	-0.05	0.039*	
		R	-3.04 ± 5.26; 0 (-18.8, 4.4)	-2.24 ± 3.59; 0 (-11.2, 1.4)	0.491	0.95	-0.87	2.78	0.310	
		V	1.67 ± 2.97; 1.44 (-6.32, 11.74)	1.95 ± 2.47; 1.62 (-2.99, 9.4)	0.689	-0.34	-1.42	0.74	0.541	
	Lateral Incisor	H	0.52 ± 1.33; 0 (-0.85, 3.92)	0.05 ± 1.77; -0.17 (-5.85, 4.16)	0.257	-0.78	-1.41	-0.16	0.017*	
		R	-3.24 ± 5.42; -0.8 (-21, 6)	-1.78 ± 3.47; 0 (-9.05, 4.2)	0.219	0.81	-0.83	2.46	0.336	
		V	1.38 ± 3.58; 1.5 (-11.3, 10.92)	2.1 ± 2.35; 1.48 (-1.14, 9.68)	0.363	0.00	-1.13	1.12	0.995	
Maxillary	1stpm	H	0 ± 0.62; 0 (-1.2, 1.78)	-0.07 ± 0.71; 0 (-2.05, 1.77)	0.700	-0.06	-0.36	0.23	0.675	
		R	-3.38 ± 9.3; 0 (-47.9, 3.7)	-0.35 ± 4.27; 0 (-7.05, 15.35)	0.112	2.39	0.49	4.29	0.017*	
		V	0.04 ± 0.86; -0.02 (-2, 1.94)	-0.13 ± 1.25; -0.14 (-3.25, 2.36)	0.540	0.07	-0.37	0.51	0.761	
	2ndpm	H	-0.3 ± 1.01; -0.12 (-4.44, 1.36)	-0.24 ± 0.68; 0 (-1.56, 1.4)	0.802	0.02	-0.25	0.29	0.866	
		R	-0.16 ± 3.54; 0 (-8.35, 11)	-0.98 ± 4.51; 0 (-12.25, 12.15)	0.441	0.69	-0.87	2.24	0.390	
		V	0.17 ± 1; 0.05 (-1.86, 2.98)	-0.18 ± 0.94; -0.1 (-2.28, 1.91)	0.165	-0.05	-0.40	0.31	0.784	
	Canine	H	0.15 ± 0.92; 0 (-2.54, 2.92)	0.16 ± 0.61; 0 (-0.85, 2.23)	0.982	-0.03	-0.38	0.31	0.852	
		R	-2.49 ± 3.7; -1.65 (-9.9, 6.4)	-3.21 ± 8.24; -1.3 (-41.5, 5.8)	0.665	1.06	-1.08	3.20	0.335	
		V	-0.07 ± 1.09; -0.04 (-3.49, 1.97)	0.17 ± 1.92; 0.15 (-3.97, 5.14)	0.550	0.39	-0.20	0.99	0.200	
	Central Incisor	H	0.19 ± 3.24; 0 (-14.54, 6.07)	0.39 ± 1.66; 0 (-5.3, 4.24)	0.760	-0.10	-0.81	0.61	0.781	
		R	-1.85 ± 4.35; -0.68 (-15.75, 9.3)	-4.06 ± 6; -3.32 (-26.5, 2.4)	0.109	-0.40	-2.09	1.29	0.646	
		V	0.38 ± 2.13; 0.21 (-4.75, 5.7)	1.15 ± 2.88; 0.52 (-2.86, 8.27)	0.246	0.58	-0.29	1.46	0.194	
	Lateral Incisor	H	0.43 ± 1.01; 0.1 (-1.1, 3.92)	0.18 ± 0.76; 0 (-1.23, 2.36)	0.265	-0.27	-0.72	0.17	0.226	
		R	-2.2 ± 3.66; -0.25 (-11.8, 4)	-3.47 ± 5.73; -1.88 (-23.5, 3.3)	0.310	-0.50	-2.25	1.25	0.578	
		V	0.09 ± 1.67; -0.07 (-4.54, 4.74)	0.58 ± 2.26; 0.17 (-3.41, 5.24)	0.342	0.65	-0.11	1.41	0.099	

* <0.05

### Vertical plane

In the adult group, the achieved vertical movements were significantly more than that predicted for the mandibular first premolars, canines, and central and lateral incisors (*p* < 0.05). Similarly, in the teenagers’ group, the achieved vertical movements were significantly more than that predicted for all assessed mandibular teeth, in addition to the maxillary central incisor (*p* < 0.05). Figure 4 and Supplementary Table 1.

When both groups were compared, there was no statistically significant difference between them in the achieved vs. predicted movements in the Vertical plane after adjusting for predicted movement (*p* > 0.05). Table 4.

### Rotations

In the adult group, the achieved rotations were significantly (*p* < 0.05) less than that predicted for the mandibular first and second premolars. Similarly, in mandibular central and lateral incisors, the achieved rotations were significantly (*p* < 0.05) less than predicted. In addition, maxillary canines, and maxillary central and lateral incisors had significantly (*p* < 0.05) less achieved rotation than predicted. In the teenagers’ group, the achieved rotations were significantly (*p* < 0.05) less than that predicted for mandibular canines, and central and lateral incisors. Similarly, in the maxillary arch, the achievement movement was significantly (*p* < 0.05) less than predicted for maxillary canines, and central and lateral incisors. Figure 4 and Supplementary Table 1.

However, when the adult and teenager groups were compared, there was no statistically significant difference between them in the achieved vs. predicted rotations after adjusting for predicted movement, except rotations were significantly (*p* = 0.017) underachieved for maxillary first premolars, more in adults than in teenagers. Table 4.

Excellent intra-examiner reliability was demonstrated for all the remeasured variables in the 10 randomly selected digital models (ICC > 0.990). Supplementary Table 2.

## Discussion

This retrospective study is the first to evaluate and compare the accuracy of tooth movement between adults and teenagers following treatment with clear aligners (Invisalign^®^ system). The null hypothesis was rejected, and tooth movement outcomes were different between teenagers and adults. Importantly both groups were comparable in the number of refinements and treatment duration.

In the transverse dimension, the within-group analysis indicated greater achieved inter-canine width compared to that predicted in the adult group only. On the other hand, the accuracy of the mandibular inter-canine width showed a statistically significant difference between the groups, it was overcorrected in adults compared to teenagers. The importance of the inter-canine width lies in its influence on the long-term stability of mandibular anterior teeth [19]. The main force application mechanism via clear aligners is the shape molding effect, which molds tooth movement according to the shape of the thermoplastic material used in aligners [9]. This implies that forces applied by clear aligners are often directed to the crowns, resulting in a default tooth movement of tipping [4]. In crowded cases treated with aligners, the primary mechanism to create space is tipping [20]. When teeth tip forward (procline), their incisal edges form an arc of greater circumference than the root apices, leading to increased inter-canine width [21].

Grunheid et al. studied the efficacy of clear aligners in increasing the transverse dimension and found a greater tendency for clear aligners to increase mandibular inter-canine width during alignment compared to fixed appliance treatment [22]. Another recent Invisalign^®^ study compared the achieved transverse dimension with that predicted in 116 patients, finding that planned expansion at the end of treatment was unpredictable, with statistically significant differences in gingival widths for canines, premolars, and molars, as well as widths at the level of cusp tips for these teeth [23]. Kravitz et al. also indicated that the Invisalign^®^ system achieves greater accuracy in mandibular anterior space closure than in relieving mandibular crowding with labial expansion alone [12]. The significantly overcorrected inter-canine width in adults compared to teenagers may be attributed to the growth of the mandible during teenage years, which often exceeds that of the maxilla, resulting in retroclination of the lower anterior teeth and dental constriction of the anterior mandible [24]. Consequently, the incisal edges form an arc with a smaller circumference than the root apices [24]. Conversely, the greater achieved inter-canine width in adults compared to predictions might be related to clinical factors, such as differences between digitally programmed and implemented interproximal reduction (IPR) [25]. The literature reports that implemented IPR is consistently lower than programmed in clear aligner therapy, potentially leading to less space for aligning incisors [25], causing flaring of the lower anterior teeth and mesialization of the dentition, contributing to increased inter-canine width.

The least predictable horizontal movement was observed in the mandibular lateral incisors in the adult group, where a greater amount of achieved tooth movement was noticed compared to that predicted. This finding contradicts the results reported by Charalampakis et al., who found no discrepancy between predicted and achieved horizontal movements in all assessed teeth in a small sample of 20 adults [8]. Conversely, in a sample of 10 adult subjects followed prospectively, the vestibulo-lingual discrepancy between planned and achieved movement for the lower incisors was non-significant [7]. The sample sizes in these studies were relatively small, which might have impacted their findings. A recent study aiming to predict lower incisor root tip movement in clear aligner therapy demonstrated that the achieved lower incisor root movement was substantially less than that displayed in the Clincheck [26]. The smaller size of the mandibular incisors, in general, might contribute to lesser control of their root movement, resulting in a greater tendency for tipping and thus greater horizontal movement. Jiang et al. utilized CBCT scans to evaluate the efficacy of clear aligners for incisor movement [20]. Their findings indicated that tipping was the most predictable movement, with a predictability rate of 72.48%. Additionally, the labial movement of the mandibular incisors showed greater efficacy (69.52%) compared to the maxillary incisors (56.16%) [20].

The significant overcorrection observed in the mandibular central and lateral incisors in adults compared to teenagers might be attributed to the presence of thinner bone in the anterior part of the mandible in adults. A cohort study that evaluated the changes in the prevalence of alveolar bone loss in a birth cohort over 8 years found that the frequency of subjects with radiographic alveolar bone loss increased with age [27]. Moreover, a positive correlation between the amount of dehiscence and the thickness of the alveolar bone in the anterior part of the mandible was previously indicated [28]. This might explain the greater tendency for the lower incisors to move forward in adults compared to teenagers. On the other hand, the lesser achieved horizontal movement for the mandibular first premolars in teenagers, compared to that predicted within the same group and to that in adults, might be attributed to the minimal planned movement for these teeth. This is because the majority of the included cases involved crowding in the lower anterior region (canine to canine area), necessitating more significant adjustments in that region rather than in the premolars.

The achieved vertical movements for most assessed teeth in adults and teenagers showed greater deviations from predictions compared to movements evaluated in other planes. Likewise, the accuracy study by Charalampakis et al. reported more notable linear changes in vertical movements than in other planes [29]. And despite their planned intrusive movements, extrusive movements were often achieved [29]. Krieger et al. also observed that the greatest discrepancies were in vertical plane movements [30]. Similarly, in this study, the amount of vertical movement was generally greater than planned, with extrusion being the dominant vertical movement.

Regarding rotational movements, it is well documented that rotating rounded teeth such as premolars and canines is more challenging than rotating incisors [9, 31]. Our study demonstrated poor predictability in rotating the mandibular premolars and incisors, as well as maxillary canines and incisors, for both adults and teenagers. Additionally, teenagers exhibited poor predictability in de-rotating their lower canines. In assessing the mean accuracy of canine rotation, Kravitz et al. reported an accuracy of 36%, which significantly improved to 43% with the implementation of IPR [11]. On the other hand, Stephens et al. found that even with the new SmartTrack aligners, the expressed canine rotation was still less than prescribed [32]. Their study indicated that vertical attachments for de-rotating teeth were associated with the least accuracy, and the efficacy was comparable between 1 and 2-week wear periods [32]. Grunheid et al. also showed that the movements with the greatest variation between achieved and predicted included the mandibular lateral, canine, and first premolar rotations [22]. In another similar study, the least accurate movements were reported for the rotation of the mandibular premolars and canines, as well as maxillary canines [29]. The variation in reported accuracy within the groups of adults and teenagers might be related to the magnitude of the rotation planned in the simulated treatment plan. It has been reported that as the staging (amount of rotation per aligner) increases, the predictability of achieving the planned rotation declines significantly [11, 33]. Simon et al. found that for premolar rotations, staging of less than 1.5° per aligner achieved an accuracy of 41.8%, while staging greater than 1.5° per aligner saw accuracy drop to 23% [33]. Therefore, the results of this study indicate that achieving angular movements is challenging in any age group. Increasing the frequency of aligner change might ensure the delivery of a more constant force and improve the accuracy in expressing the planned rotation [4].

### Strengths, limitations, and clinical significance

The comparison of accuracy in achieving simulated tooth movement focused on the magnitude of the difference between predicted and achieved outcomes rather than percentages. This approach allows for a more comprehensive and detailed evaluation of the studied tooth movements [29]. On the other hand, the clinical significance of this study lies in its distinction as the first to compare the accuracy of achieved movements using clear aligners between adults and teenagers. This paves the way for future trials aimed at comparing the efficacy of clear aligners in treating different age groups with various malocclusions.

Similar to previous studies that utilized downloaded models from ClinCheck software to assess accuracy, it’s important to note that the desired final tooth position may not align precisely with the prescribed position in the simulated treatment plan (ClinCheck) [10]. This discrepancy arises because the simulated treatment plan represents the force system (with overengineering) rather than the exact predicted final tooth position [34].

The primary limitations of this study include its retrospective nature and the potential for selection bias. To mitigate this, patients were randomly selected from the database. Other Limitations might be related to patients’ compliance in wearing the clear aligners as prescribed, the collected clinical variables that are related to the number of refinements per group, and the duration of treatment were comparable between teenagers and adults indicating possible good compliance with treatment within the groups. Since the sample was drawn exclusively from patients treated with Invisalign^®^, the findings may not be generalizable to other aligner systems or practitioners. Furthermore, variations in the use of attachments, IPR, and auxiliaries could have influenced the accuracy of tooth movement but were not accounted for due to the retrospective nature of the study.

## Conclusion

The study revealed significant differences in tooth movement outcomes between adults and teenagers. Specifically, inter-canine width in the transverse plane which was overcorrected in the adults’ group compared to teenagers. In the horizontal plane, the mandibular central and lateral incisors exhibited significantly greater over-correction in adults compared to teenagers. However, deviations in the vertical plane and rotations were comparable between the two age groups.

## Electronic supplementary material

Below is the link to the electronic supplementary material.

**Supplementary Material 1**: **Supplementary table 1**. Descriptive statistics for the various predicted and achieved movements for adults and teenagers.

**Supplementary Material 2**: **Supplementary table 2**. Intra-examiner reliability.

## Data Availability

No datasets were generated or analysed during the current study.

## Abbreviations

AVDM: Achieved virtual dental model
CAD/CAM: Computer-Aided Design (CAD) and Computer-Aided Manufacturing (CAM)
IPR: Interproximal reduction
PVDM: Predicted virtual dental model

## References

[1] Hennessy J, Al-Awadhi EA. Clear aligners generations and orthodontic tooth movement. J Orthod. 2016;43(1):68–76. 25939782 10.1179/1465313315Y.0000000004

[2] Wong BH. Invisalign a to Z. Am J Orthod Dentofac Orthop. 2002;121(5):540–1. 10.1067/mod.2002.123036 12045774

[3] Jia L, Huo S, Wang Y, Hu S, Zhao J, Fan Y, et al. The effects of lingual buttons, precision cuts, and patient-specific attachments during maxillary molar distalization with clear aligners: comparison of finite element analysis. Am J Orthod Dentofac Orthop. 2023;163(1):e1–12. 10.1016/j.ajodo.2022.10.010 36435687

[4] Upadhyay M, Arqub SA. Biomechanics of clear aligners: hidden truths & first principles. J World Fed Orthod. 2022;11(1):12–21. 34965910 10.1016/j.ejwf.2021.11.002

[5] Weir T. Clear aligners in orthodontic treatment. Aust Dent J. 2017;62:58–62. 28297094 10.1111/adj.12480

[6] Wheeler TT. Orthodontic clear aligner treatment. Semin Orthod. Elsevier; 2017.

[7] Bilello G, Franchi L, Graci G, Masucci C, Baccetti T. Accuracy evaluation of orthodontic movements with aligners: a prospective observational study. Prog Orthod. 2022;23(1):1–8. 35399128 10.1186/s40510-022-00406-7 PMC8995220

[8] Charalampakis O, Iliadi A, Ueno H, Oliver DR, Kim KB. Accuracy of clear aligners: a retrospective study of patients who needed refinement. Am J Orthod Dentofac Orthop. 2018;154(1):47–54. 10.1016/j.ajodo.2017.11.028 29957318

[9] Rossini G, Parrini S, Castroflorio T, Deregibus A, Debernardi CL. Efficacy of clear aligners in controlling orthodontic tooth movement: a systematic review. Angle Orthod. 2015;85(5):881–9. 25412265 10.2319/061614-436.1 PMC8610387

[10] Haouili N, Kravitz ND, Mazur M, Vaid NR. Has Invisalign improved? A prospective follow-up study on the efficacy of tooth movement with Invisalign. Am J Orthod Dentofac Orthop. 2020;158(3):420–5. 10.1016/j.ajodo.2019.12.015 32620479

[11] Kravitz ND, Kusnoto B, Agran B, Viana G. Influence of attachments and interproximal reduction on the accuracy of canine rotation with Invisalign: a prospective clinical study. Angle Orthod. 2008;78(4):682–7. 18302468 10.2319/0003-3219(2008)078[0682:IOAAIR]2.0.CO;2

[12] Kravitz ND, Kusnoto B, BeGole E, Obrez A, Agran B. How well does Invisalign work? A prospective clinical study evaluating the efficacy of tooth movement with Invisalign. Am J Orthod Dentofac Orthop. 2009;135(1):27–35. 10.1016/j.ajodo.2007.05.018 19121497

[13] Djeu G, Shelton C, Maganzini A. Outcome assessment of Invisalign and traditional orthodontic treatment compared with the American Board of Orthodontics objective grading system. Am J Orthod Dentofac Orthop. 2005;128(3):292–8. 10.1016/j.ajodo.2005.06.002 16168325

[14] Gu J, Tang JS, Skulski B, Fields HW, Beck FM, Firestone AR. Evaluation of Invisalign treatment effectiveness and efficiency compared with conventional fixed appliances using the peer Assessment Rating index. Am J Orthod Dentofac Orthop. 2017;151(2):259–66. 10.1016/j.ajodo.2016.06.041 28153154

[15] Hennessy J, Garvey T, Al-Awadhi EA. A randomized clinical trial comparing mandibular incisor proclination produced by fixed labial appliances and clear aligners. Angle Orthod. 2016;86(5):706–12. 27571371 10.2319/101415-686.1 PMC8600838

[16] Borda AF, Garlock FL, Swanson WD, Wang Y, Liu S. Outcome assessment of orthodontic clear aligner vs fixed appliance treatment in a teenage population with mild malocclusions. Angle Orthod. 2020;90(4):485–90. 33378505 10.2319/122919-844.1 PMC8028462

[17] Harandi MT, Alikhani M, Mahdizi M, Wiese M, Alyami B, Baek E, et al. Assessment of clear aligner accuracy of 2 clear aligners systems. Am J Orthod Dentofac Orthop. 2023;164(6):793–804. 10.1016/j.ajodo.2023.05.028 37498253

[18] Årtun J, Garol JD, Little RM. Long-term stability of mandibular incisors following successful treatment of Class II, Division 1, malocclusions. Angle Orthod. 1996;66(3):229–38. 8805919 10.1043/0003-3219(1996)066<0229:LTSOMI>2.3.CO;2

[19] Jiang T, Chen X, Hu W, Zhong Z, Zou W. A cone-beam computed tomographic study evaluating the efficacy of incisor movement with clear aligners: Assessment of incisor pure tipping, controlled tipping, translation, and torque. Am J Orthod Dentofac Orthop. 2021;159(5):635–43. 10.1016/j.ajodo.2019.11.025 33583693

[20] Popp TW, Gooris CG, Nanda RS. Nonsurgical treatment for a class III dental relationship: a case report. Am J Orthod Dentofac Orthop. 1993;103(3):203–11. 10.1016/0889-5406(93)70001-5 8456776

[21] Grünheid T, Loh C, Larson BE. Effect of clear aligner therapy on the buccolingual inclination of mandibular canines and the intercanine distance. Angle Orthod. 2016;86(1):10–6. 26000701 10.2319/012615-59.1 PMC8603951

[22] Solano-Mendoza B, Sánchez-Molina L, Madrigal C, Villalobos C, Salcedo JM, Oteo-Calatayud F. How effective is the Invisalign^®^ system in expansion movement with Ex30′ aligners? Clin Oral Investig. 2017;21:1475–84. 27435982 10.1007/s00784-016-1908-y

[23] Björk A, Palling M. Adolescent age changes in sagittal jaw relation, alveolar prognathy, and incisal inclination. Acta Odontol Scand. 1955;12(3–4):201–32. 14375891 10.3109/00016355509028164

[24] Hariharan A, Moosazadeh S, Mitha A, Masucci C. Evaluation of interproximal reduction in individual teeth, and full arch assessment in clear aligner therapy: digital planning versus 3D model analysis after reduction. Prog Orthod. 2022;23(1):9. 35254555 10.1186/s40510-022-00403-w PMC8901911

[25] Smith JM, Lin R, Dominguez GC, Wheatley DC, Tuncay OC. Predictability of lower incisor tip using clear aligner therapy. Prog Orthod. 2022;23(1):37. 36336726 10.1186/s40510-022-00433-4 PMC9637687

[26] Aass AM, Tollefsen T, Gjermo P. A cohort study of radiographic alveolar bone loss during adolescence. J Clin Periodontol. 1994;21(2):133–8. 8144733 10.1111/j.1600-051x.1994.tb00291.x

[27] Siriwat PP, Jarabak JR. Malocclusion and facial morphology: is there a relationship? An epidemiologic study. Angle Orthod. 1985;55(2):127–38. 3874569 10.1043/0003-3219(1985)055<0127:MAFMIT>2.0.CO;2

[28] Krieger E, Seiferth J, Marinello I, Jung BA, Wriedt S, Jacobs C et al. Invisalign^®^ treatment in the anterior region. J Orofac Orthop. 2012:1–12. 10.1007/s00056-012-0097-9 22890691

[29] Papadimitriou A, Mousoulea S, Gkantidis N, Kloukos D. Clinical effectiveness of Invisalign^®^ orthodontic treatment: a systematic review. Prog Orthod. 2018;19(1):1–24. 30264270 10.1186/s40510-018-0235-z PMC6160377

[30] Stephens C, Berk NW, Kloth D, Sateesh A, Seagle B. Clinical expression of programmed mandibular canine rotation using various attachment protocols and 1- vs 2-week wear protocols with Invisalign SmartTrack aligners: a retrospective cohort study. Am J Orthod Dentofac Orthop. 2022;162(3):e103–15. 10.1016/j.ajodo.2022.06.015 35835703

[31] Simon M, Keilig L, Schwarze J, Jung BA, Bourauel C. Treatment outcome and efficacy of an aligner technique–regarding incisor torque, premolar derotation and molar distalization. BMC Oral Health. 2014;14:1–7. 24923279 10.1186/1472-6831-14-68 PMC4068978

[32] Glaser BJ. The Insider’s Guide to Invisalign Treatment: A Step-By-Step Guide to Assist You with Your ClinCheck Treatment Plans. 3L Publishing; 2017.

[33] Papadimitriou A, Mousoulea S, Gkantidis N, Kloukos D. Clinical effectiveness of Invisalign® orthodontic treatment: a systematic review. Progr Orthodont 2018;19:1–24. 10.1186/s40510-018-0235-z PMC6160377 30264270

[34] Stephens C, Weir T, Llewellyn S, Freer E, Kerr B. Clinical expression of programmed mandibular canine rotation using various attachment protocols and 1-vs 2-week wear protocols with Invisalign SmartTrack aligners: A retrospective cohort study. Am J Orthodont Dentofac Orthop 2022;162:e103–e115. 10.1016/j.ajodo.2022.06.015 35835703

