# Supplementary material for: Age-stratified assessment of orthodontic tooth movement outcomes with clear aligners
Source: Prog Orthod. 2024 Nov 11;25:43. doi: 10.1186/s40510-024-00542-2 (PMC11551086; doi:10.1186/s40510-024-00542-2)
Supplement: Supplementary file 1 — Supplementary Material 1: Supplementary table 1. Descriptive statistics for the various predicted and achieved movements for adults and teenagers. [file 40510_2024_542_MOESM1_ESM.docx]

**Supplementary table 1-** Descriptive statistics for the various predicted and achieved movements for adults and teenagers

| **Arch** | **tooth** | **Plane** | **Predicted**  **mean ± standard deviation;**  **median (minimum, maximum)** | **Achieved**  **mean ± standard deviation;**  **median (minimum, maximum)** | **Accuracy**  **mean ± standard deviation;**  **median (minimum, maximum)** | **P-value** |
| --- | --- | --- | --- | --- | --- | --- |
| **Adults** | | | | | | |
| **Mandibular** | **1stpm** | **H** | 0.63 ± 1.34;  0.55 (-3.37, 2.88) | 0.69 ± 1.22;  0.28 (-2.84, 3.23) | 0.06 ± 0.8;  0.03 (-1.25, 2.35) | 0.668 |
|  |  | **R** | **2.91 ± 5.21;**  **0 (0, 24.8)** | **1.53 ± 3.16;**  **0 (0, 14.5)** | **-1.38 ± 3.45;**  **0 (-11.8, 3.65)** | **0.037** |
|  |  | **V** | **-0.19 ± 1.3;**  **-0.28 (-2.19, 3.27)** | **0.3 ± 0.9;**  **0 (-1.29, 3.12)** | **0.49 ± 0.95;**  **0.61 (-1.77, 2.16)** | **0.009** |
|  | **2ndpm** | **H** | 0.79 ± 1.19;  0.54 (-1.27, 3.85) | 0.85 ± 0.89;  0.7 (-0.94, 2.63) | 0.05 ± 1.04;  -0.01 (-3.71, 2.6) | 0.787 |
|  |  | **R** | **1.87 ± 2.64;**  **0 (0, 7.5)** | **0.81 ± 1.6;**  **0 (0, 7.25)** | **-1.06 ± 2.83;**  **0 (-7.5, 7.25)** | **0.049** |
|  |  | **V** | 0.13 ± 0.73;  0 (-1.29, 2.3) | 0.27 ± 0.59;  0.16 (-0.36, 2.34) | 0.14 ± 0.63;  0.05 (-1.02, 1.75) | 0.226 |
|  | **Canine** | **H** | 0.14 ± 1.75; 0 (-5.21, 3.53) | 0.52 ± 1.3; 0.06 (-3.94, 3.64) | 0.39 ± 1.37; 0 (-1.83, 5.28) | 0.131 |
|  |  | **R** | 6.82 ± 9.56;  3.53 (0, 33.45) | 4.08 ± 5.89;  1 (0, 21.25) | -2.74 ± 7.9;  0 (-33, 9.5) | 0.067 |
|  |  | **V** | **-1.11 ± 1.64;**  **-1.08 (-3.76, 2.21)** | **0 ± 1.33;**  **0 (-2.63, 3.67)** | **1.12 ± 1.77;**  **0.95 (-2.42, 7.44)** | **0.002** |
|  | **Central**  **Incisor** | **H** | 0.3 ± 2.28;  0.12 (-5.96, 4.7) | 0.73 ± 2.05;  0.68 (-6.1, 5.09) | 0.43 ± 1.57;  0 (-3.2, 4.57) | 0.149 |
|  |  | **R** | **5.98 ± 8.29;**  **0.5 (0, 29)** | **2.94 ± 5.72;**  **0 (0, 21.5)** | **-3.04 ± 5.26;**  **0 (-18.8, 4.4)** | **0.004** |
|  |  | **V** | **-1.62 ± 2.08;**  **-1.93 (-5.72, 2.97)** | **0.05 ± 2.11;**  **-0.12 (-3.36, 6.02)** | **1.67 ± 2.97;**  **1.44 (-6.32, 11.74)** | **0.005** |
|  | **Lateral Incisor** | **H** | **0.55 ± 2.64;**  **0 (-5.37, 5.81)** | **1.07 ± 2.33;**  **0.62 (-5.61, 5.96)** | **0.52 ± 1.33;**  **0 (-0.85, 3.92)** | **0.042** |
|  |  | **R** | **5.78 ± 6.55;**  **3.5 (0, 22)** | **2.55 ± 4.56;**  **0.5 (0, 20)** | **-3.24 ± 5.42;**  **-0.8 (-21, 6)** | **0.003** |
|  |  | **V** | **-1.77 ± 2.59;**  **-1.67 (-5.54, 5.66)** | **-0.39 ± 2.43;**  **-0.28 (-5.64, 5.45)** | **1.38 ± 3.58;**  **1.5 (-11.3, 10.92)** | **0.043** |
| **Maxillary** | **1stpm** | **H** | 0.58 ± 0.99;  0.16 (-1.75, 2.72) | 0.58 ± 0.97;  0.27 (-1.81, 2.36) | 0 ± 0.62;  0 (-1.2, 1.78) | 0.976 |
|  |  | **R** | 5.32 ± 9.89;  1.95 (0, 50.9) | 1.93 ± 2.42;  0.75 (0, 8.45) | -3.38 ± 9.3;  0 (-47.9, 3.7) | 0.056 |
|  |  | **V** | 0.14 ± 1.2;  0.08 (-2.05, 2.33) | 0.17 ± 0.83;  0.28 (-2.09, 1.75) | 0.04 ± 0.86;  -0.02 (-2, 1.94) | 0.819 |
|  | **2ndpm** | **H** | 0.76 ± 1.31;  0.46 (-1.45, 4.98) | 0.46 ± 0.78;  0.25 (-1.17, 2.38) | -0.3 ± 1.01;  -0.12 (-4.44, 1.36) | 0.116 |
|  |  | **R** | 1.63 ± 2.85; 0 (0, 9.15) | 1.47 ± 2.63; 0 (0, 11) | -0.16 ± 3.54; 0 (-8.35, 11) | 0.802 |
|  |  | **V** | 0.02 ± 0.97; -0.12 (-1.62, 2.66) | 0.19 ± 0.59; 0.25 (-1.17, 1.59) | 0.17 ± 1; 0.05 (-1.86, 2.98) | 0.354 |
|  | **canine** | **H** | 0.15 ± 1.32; 0 (-2.76, 2.79) | 0.3 ± 1.11; 0 (-2.54, 2.98) | 0.15 ± 0.92; 0 (-2.54, 2.92) | 0.373 |
|  |  | **R** | **5.16 ± 6.17; 3.35 (0, 24)** | **2.58 ± 4.5; 0 (0, 19)** | **-2.49 ± 3.7; -1.65 (-9.9, 6.4)** | **0.001** |
|  |  | **V** | 0.44 ± 1.55; 0.8 (-2.8, 3.24) | 0.37 ± 1.21; 0.68 (-2.77, 2.72) | -0.07 ± 1.09; -0.04 (-3.49, 1.97) | 0.731 |
|  | **Central Incisor** | **H** | -0.03 ± 3.37; 0 (-5.05, 15.65) | 0.15 ± 1.58; 0 (-4.84, 5.14) | 0.19 ± 3.24; 0 (-14.54, 6.07) | 0.756 |
|  |  | **R** | **5.68 ± 6.64; 3.4 (0, 23.05)** | **3.83 ± 4.45; 1.9 (0, 17.15)** | **-1.85 ± 4.35; -0.68 (-15.75, 9.3)** | **0.027** |
|  |  | **V** | 0.25 ± 2.23; 0.5 (-3.88, 4.87) | 0.63 ± 1.65; 0.72 (-3.33, 5.3) | 0.38 ± 2.13; 0.21 (-4.75, 5.7) | 0.335 |
|  | **Lateral**  **Incisor** | **H** | **-0.09 ± 1.92; 0 (-4.82, 5.67)** | **0.34 ± 1.73; 0 (-4.66, 5.24)** | **0.43 ± 1.01; 0.1 (-1.1, 3.92)** | **0.025** |
|  |  | **R** | **5.68 ± 6.98; 3.4 (0, 29.1)** | **3.49 ± 5.47; 1.12 (0, 22.5)** | **-2.2 ± 3.66; -0.25 (-11.8, 4)** | **0.003** |
|  |  | **V** | 0.3 ± 2.31; 0.82 (-5.69, 4.69) | 0.39 ± 1.53; 0.6 (-2.6, 4.62) | 0.09 ± 1.67; -0.07 (-4.54, 4.74) | 0.774 |
| **Teenagers** | | | | | | |
| **Mandibular** | **1stpm** | **H** | **0.57 ± 1.27; 0.07 (-2.62, 2.84)** | **0.3 ± 1.06; 0.26 (-3.47, 2.45)** | **-0.27 ± 0.6; -0.06 (-1.71, 0.52)** | **0.022** |
|  |  | **R** | 5.16 ± 5.9; 4.08 (0, 19.2) | 4.19 ± 6.24; 2.28 (0, 25.5) | -0.97 ± 4.4; 0 (-14.1, 12.7) | 0.237 |
|  |  | **V** | **-0.43 ± 1.03; -0.31 (-2.7, 1.14)** | **0.24 ± 0.91; 0.16 (-1.36, 3.21)** | **0.67 ± 1.4; 0.41 (-2.28, 4.04)** | **0.014** |
|  | **2ndpm** | **H** | 0.49 ± 1.08; 0 (-1.72, 3.28) | 0.44 ± 0.58; 0.31 (0, 2.64) | -0.04 ± 0.68; 0 (-1.44, 1.72) | 0.728 |
|  |  | **R** | 8.62 ± 12.36; 3.95 (0, 42.8) | 6.41 ± 8.56; 3.92 (0, 29.9) | -2.21 ± 8.15; 0 (-31.35, 9.75) | 0.148 |
|  |  | **V** | **-0.09 ± 0.92; -0.06 (-1.73, 1.54)** | **0.34 ± 0.63; 0.38 (-0.72, 1.85)** | **0.43 ± 0.96; 0.36 (-1.42, 2.64)** | **0.021** |
|  | **Canine** | **H** | -0.33 ± 1.8; 0 (-5.46, 3.17) | 0.06 ± 1.8; 0 (-4.81, 7.17) | 0.4 ± 1.3; 0 (-2, 4.06) | 0.105 |
|  |  | **R** | **11.74 ± 12.01; 10.45 (0, 39.5)** | **7.08 ± 7.2; 5.75 (0, 28.3)** | **-4.66 ± 9.12; -2.45 (-34.5, 6.1)** | **0.009** |
|  |  | **V** | **-2.13 ± 1.75; -2.16 (-5.66, 0.98)** | **-0.58 ± 1.69; -0.67 (-3.41, 4.7)** | **1.55 ± 1.95; 1.13 (-0.92, 9.14)** | **0.000** |
|  | **Central**  **Incisor** | **H** | -0.7 ± 2.77; 0 (-6.23, 7.95) | -0.52 ± 1.62; 0 (-5.61, 1.86) | 0.18 ± 2.02; 0 (-6.5, 4.31) | 0.635 |
|  |  | **R** | **6.4 ± 7.37; 4.1 (0, 23.85)** | **4.16 ± 6.7; 0.73 (0, 25.25)** | **-2.24 ± 3.59; 0 (-11.2, 1.4)** | **0.002** |
|  |  | **V** | **-2.44 ± 2.56; -2.24 (-7, 2.8)** | **-0.49 ± 2.2; -0.77 (-4.24, 5.41)** | **1.95 ± 2.47; 1.62 (-2.99, 9.4)** | **0.000** |
|  | **Lateral Incisor** | **H** | -0.26 ± 2.48; 0 (-6.24, 6.75) | -0.21 ± 1.52; 0 (-5.33, 1.99) | 0.05 ± 1.77; -0.17 (-5.85, 4.16) | 0.869 |
|  |  | **R** | **4.57 ± 5.55; 4.6 (0, 24.45)** | **2.79 ± 3.99; 1.02 (0, 18.25)** | **-1.78 ± 3.47; 0 (-9.05, 4.2)** | **0.009** |
|  |  | **V** | **-2.6 ± 2.23; -2.17 (-7.22, 0.98)** | **-0.5 ± 1.94; -0.54 (-3.41, 5.4)** | **2.1 ± 2.35; 1.48 (-1.14, 9.68)** | **0.000** |
| **Maxillary** | **1stpm** | **H** | 0.59 ± 1.16; 0.28 (-1.77, 3.21) | 0.52 ± 0.93; 0.18 (-1.1, 3.22) | -0.07 ± 0.71; 0 (-2.05, 1.77) | 0.592 |
|  |  | **R** | 4.46 ± 6.19; 1.62 (0, 22) | 4.11 ± 5.49; 1.62 (0, 16.2) | -0.35 ± 4.27; 0 (-7.05, 15.35) | 0.657 |
|  |  | **V** | 0.59 ± 1.31; 0.65 (-2.18, 3.04) | 0.45 ± 1.2; 0.51 (-2.11, 2.82) | -0.13 ± 1.25; -0.14 (-3.25, 2.36) | 0.560 |
|  | **2ndpm** | **H** | 0.71 ± 1.03; 0.38 (-1.4, 2.68) | 0.46 ± 0.68; 0.36 (-0.74, 2) | -0.24 ± 0.68; 0 (-1.56, 1.4) | 0.060 |
|  |  | **R** | 4.84 ± 7.98; 1.5 (0, 37.6) | 3.87 ± 5.54; 2.25 (0, 26.9) | -0.98 ± 4.51; 0 (-12.25, 12.15) | 0.246 |
|  |  | **V** | 0.46 ± 1.08; 0.49 (-2.04, 2.51) | 0.28 ± 0.89; 0.38 (-1.63, 1.88) | -0.18 ± 0.94; -0.1 (-2.28, 1.91) | 0.301 |
|  | **canine** | **H** | 0.03 ± 1.02; 0 (-2.96, 1.96) | 0.19 ± 0.98; 0 (-2.42, 2.42) | 0.16 ± 0.61; 0 (-0.85, 2.23) | 0.166 |
|  |  | **R** | **7.97 ± 9.14; 4.75 (0, 41.5)** | **4.76 ± 5.38; 2.78 (0, 18.85)** | **-3.21 ± 8.24; -1.3 (-41.5, 5.8)** | **0.041** |
|  |  | **V** | 0.68 ± 1.81; 0.86 (-3.09, 4.19) | 0.86 ± 1.45; 1.07 (-2.38, 3.23) | 0.17 ± 1.92; 0.15 (-3.97, 5.14) | 0.624 |
|  | **Central Incisor** | **H** | -0.42 ± 1.93; 0 (-4.64, 6.3) | -0.03 ± 1.41; 0 (-5.72, 2.44) | 0.39 ± 1.66; 0 (-5.3, 4.24) | 0.209 |
|  |  | **R** | **9.03 ± 8.52; 7.43 (0, 36.5)** | **4.97 ± 5.07; 3.28 (0, 17.05)** | **-4.06 ± 6; -3.32 (-26.5, 2.4)** | **0.001** |
|  |  | **V** | **0.03 ± 2.28; 0.58 (-4.02, 4.14)** | **1.18 ± 1.84; 1.19 (-1.82, 6.3)** | **1.15 ± 2.88; 0.52 (-2.86, 8.27)** | **0.037** |
|  | **Lateral**  **Incisor** | **H** | -0.22 ± 1.49; 0 (-3.84, 3.54) | -0.04 ± 1.73; 0 (-5.03, 3.06) | 0.18 ± 0.76; 0 (-1.23, 2.36) | 0.215 |
|  |  | **R** | **7.31 ± 7.26; 5.62 (0, 27.15)** | **3.84 ± 4.6; 3.1 (0, 21.55)** | **-3.47 ± 5.73; -1.88 (-23.5, 3.3)** | **0.002** |
|  |  | **V** | 0.56 ± 1.84; 0.77 (-3.2, 3.82) | 1.14 ± 1.81; 1.04 (-2.4, 5.32) | 0.58 ± 2.26; 0.17 (-3.41, 5.24) | 0.171 |
