# Supplementary material for: Age-stratified assessment of orthodontic tooth movement outcomes with clear aligners
Source: Prog Orthod. 2024 Nov 11;25:43. doi: 10.1186/s40510-024-00542-2 (PMC11551086; doi:10.1186/s40510-024-00542-2)
Supplement: Supplementary file 2 — Supplementary Material 2: Supplementary table 2. Intra-examiner reliability. [file 40510_2024_542_MOESM2_ESM.docx]

**Supplementary Table 2-**

**A: Intra-examiner reliability for 5 achieved models**

| **Variable** | **ICC** | **CI** |
| --- | --- | --- |
| **hor-mx** | 0.99982233 | [1.00; 1.00] |
| **vert-mx** | 0.98263104 | [0.97; 0.99] |
| **rotation-mx** | 0.99964691 | [1.00; 1.00] |
| **hor-mn** | 0.99924069 | [1.00; 1.00] |
| **vert-mn** | 0.99798442 | [1.00; 1.00] |
| **rotation-mx** | 0.99901926 | [1.00; 1.00] |

B- **Intra-examiner reliability for 5 Predicted models**

| **Variable** | **ICC** | **CI** |
| --- | --- | --- |
| **hor-mx** | 0.98783459 | [0.98; 0.99] |
| **vert-mx** | 0.99967622 | [1.00; 1.00] |
| **rotation-mx** | 0.9992953 | [1.00; 1.00] |
| **hor-mn** | 0.99867844 | [1.00; 1.00] |
| **vert-mn** | 0.99930355 | [1.00; 1.00] |
| **rotation-mx** | 0.9997847 | [1.00; 1.00] |
